# Neurovascular Interactions in the Development of the Vasculature

**DOI:** 10.3390/life13010042

**Published:** 2022-12-23

**Authors:** Kazuhiro Takara, Yumiko Hayashi-Okada, Hiroyasu Kidoya

**Affiliations:** 1 Department of Integrative Vascular Biology, Faculty of Medical Sciences, University of Fukui, Fukui 910-1193, Japan; 2 Tenure-Track Program for Innovative Research, University of Fukui, Fukui 910-1193, Japan

**Keywords:** blood vessels, vascular patterning, angiogenesis, vasculogenesis, endothelial cells, tumour vessels, tissue repair, angiocrine factors

## Abstract

Vertebrates have developed a network of blood vessels and nerves throughout the body that enables them to perform complex higher-order functions and maintain homeostasis. The 16th-century anatomical text ‘De humani corporis fabrica’ describes the networks of blood vessels and nerves as having a branching pattern in which they are closely aligned and run parallel one to another. This close interaction between adjacent blood vessels and nerves is essential not only for organogenesis during development and repair at the time of tissue damage but also for homeostasis and functional expression of blood vessels and nerves. Furthermore, it is now evident that disruptions in neurovascular interactions contribute to the progression of various diseases including cancer. Therefore, we highlight recent advances in vascular biology research, with a particular emphasis on neurovascular interactions.

## 1. Introduction

The formation of blood vessels, which occurs during various phases of development, tissue repair, and disease progression, is facilitated by the interactions between diverse cell populations in the tissue microenvironment. The supply of oxygen, nutrients, signalling molecules, and metabolites and the elimination of unwanted waste products by functional blood vessels are essential for tissue growth and homeostasis. The intima of blood vessels is covered by a monolayer of endothelial cells that junction with each other to form the surface facing the lumen. Its outer tunica media are composed primarily of pericytes and smooth muscle cells and are supported by extracellular matrix proteins, including elastic and collagen fibres. The tunica adventitia is the outermost layer of the blood vessels and contains a variety of cell types, including fibroblasts, macrophages, and adipocytes [1].

In addition to acting as a barrier to regulate solute exchange between the vessel and the surrounding tissue, endothelial cells that line the lumen of blood vessels contribute to homeostasis by regulating vasoconstriction, haemostasis, coagulation, inflammation, and other biological reactions [2,3]. In addition, endothelial cells produce angiocrine factors that regulate angiogenesis, as well as the growth, differentiation, and repair of tissues; signals from the tissue are fed back to form an appropriate vascular network [4]. Furthermore, the interaction between nerves and blood vessels via angiocrine factors is crucial, as it is involved in the development of various diseases, such as cancer.

Two mechanisms govern the formation of blood vessels during development, vasculogenesis and angiogenesis [5]. In vasculogenesis, blood vessels are formed de novo from endothelial progenitor cells called angioblasts, which are derived from the mesoderm. In contrast, angiogenesis involves growth and elongation of new sprouts from the existing vascular network [6]. In vascular sprouting, tip cells migrate in response to angiogenic signals such as vascular endothelial growth factor (VEGF). Subsequently, stalk cells that follow the tip cells proliferate to support the vessel growth [7]. Angiogenesis differs from vasculogenesis in that it not only occurs during embryogenesis, but is also involved in wound repair, tissue regeneration, and diseases such as cancer. Through vascular remodelling, the primary vascular plexus develops into a hierarchical network of arteries, veins, and capillaries [8].

Interactions with blood vessels and nerves have been shown to be not only essential for vascular formation and patterning but also to be actively involved in tissue repair and pathological progression. This article reviews the current state of knowledge regarding the interaction between blood vessels and nerves in homeostasis and disease formation, including (1) vascular formation and neural guidance molecules, (2) formation of arteries and veins through nerve–vessel interaction, and (3) nerve–vessel interaction in tumour growth and tissue repair.

## 2. Molecules for Vascular Formation and Neural Guidance

Blood vessel formation can be classified into two types: vasculogenesis and angiogenesis. Vasculogenesis is the formation of blood vessels that is induced during embryonic development, while angiogenesis branches off existing blood vessels. During vasculogenesis, vascular endothelial cells differentiated from mesodermal tissue connect with each other to form a lumen. In addition, pericytes in capillaries and small veins, as well as vascular smooth muscle cells in arteries and veins, cover vascular endothelial cells and form structurally stable vessels. Vascular endothelial cell development from the mesoderm and the subsequent proliferation and survival of vascular endothelial cells are induced primarily by VEGF, which activates its receptor, VEGFR2. Tissue-specific angiogenesis occurs through signalling mediated not only by VEGF but also by a variety of other molecules and their interactions with the surrounding cells during angiogenesis. It is well known that blood vessels and nerves have quite close anatomical organisation, and that the same molecules are involved in the formation of their networks. Therefore, in this section, we focus on the coordinated network of blood vessels and nerves in angiogenesis and introduce molecules called ‘vascular-neural guidance molecules’.

**Figure 1:**
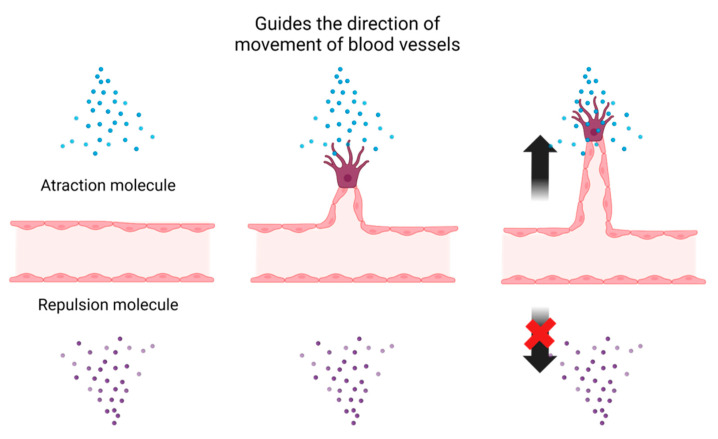
Determination of the direction of angiogenesis by guidance molecules. Guidance molecules have either attracting or repelling properties. This property allows vessels to extend and reach the appropriate location, forming a tissue-specific vascular network. Blood vessels are regulated to extend in the direction of guidance molecules of attractive properties, but not in the direction of guidance molecules of repulsive properties. Created with BioRender.com.

Vascular-neural guidance molecules have been identified as axon guidance factors in neural network formation. The attraction or repulsion of axon guidance factors with nerve axons resulting in the establishment of neural networks at appropriate locations (Figure 1). Vessels, such as nerve axons, are modulated by numerous guidance molecules to form vascular networks by extending and reaching tissues in need of the blood vessels. Semaphorin3E (Sema3E) and its receptor plexinD1 are known to be neuroaxonal guidance molecules that regulate embryonic neurogenesis. In mice deficient in either molecule, the disorganisation of the vascular network occurs during embryonic development [9]. Slit and its receptor, Robo (especially robo4 expressed in vascular endothelial cells), have been reported to contribute to axon growth and network formation via repulsive signals [10]. Robo4 has been reported to contribute to endothelial cell migration, proliferation, and angiogenesis and has also been shown to inhibit pathological angiogenesis by suppressing VEGFR2 activation and stabilising vascular endothelial cells. Sonic hedgehog (Shh) is an important morphogen involved in embryonic morphogenesis, emanating from the source in a concentration gradient. Vascular regulation has been reported, such as the absence of vascular branching in the lungs during the development of Shh-null mutants [11] and the involvement of Shh overexpression in neuroectodermal vascularisation in the dorsal neural tube [12].

Recent studies have reported that neural guidance molecules such as Neurotrophin-3 [13], Semaphorin 7A [14], and Gremlin-1 [15] are important molecules in the endothelial to mesenchymal transition (EndMT). This suggests that vascular neural guidance molecules not only contribute to the organised formation of blood vessels but also influence the differentiation of vascular endothelial cells. Although the formation of blood vessels and nerves is regulated by the same molecules, how they can be elaborately organised throughout the body, involving organ-specific vascular formation mechanisms, is a question that remains to be elucidated.

## 3. Formation of Arteries and Veins by Nerve–Vessel Interaction

### 3.1. Regulation of Arterial Patterning

**Figure 2:**
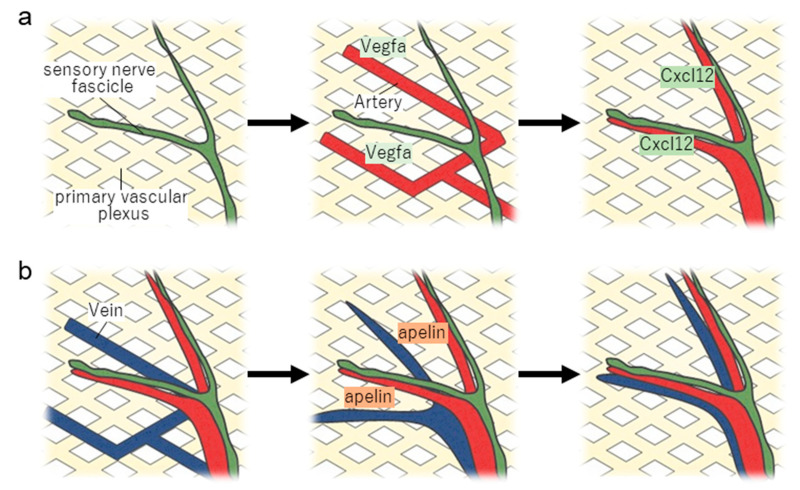
Neurovascular interactions induce the formation of arteries and veins. (**a**) Peripheral sensory nerve fascicles invading the primary vascular plexus produce VEGFA, which stimulates the arterialisation of nearby capillary. Parallel arterial–vein branching patterns are completed when Cxcl12 wires differentiated arteries into the vicinity of sensory nerve fascicles. (**b**) A part of the primitive vascular plexus then develops venous differentiation, and veins migrate towards the arteries by the effect of apelin produced by the arteries on APJ expressed in the veins. The veins mature as they migrate, and eventually, a parallel structure of nerves, arteries, and veins is established.

The immature vascular plexus formed by angiogenesis and vasculogenesis matures into a hierarchical vascular structure composed of arteries, veins, and capillaries through vascular remodelling. Sensory nerve fibres have been shown to regulate the formation of blood vessels and branching patterns in foetal mouse skin vascular remodelling. In the process of skin vascular and neural network formation in foetal mice, an immature vascular plexus is initially formed around embryonic day 11 via angiogenesis and vasculogenesis. On embryonic day 13, the peripheral nervous system sensory nerve fascicles extend their axons and form a neural network within the vascular plexus. Subsequently, arteriolar vessels develop in parallel with the branching pattern of sensory nerve fascicles [16]. In this instance, peripheral nerve fascicles and arterial blood vessels do not adhere to one another but rather form structures that run together at regular intervals. Neurogenin1/Neurogenin2 double-knockout animals, in which sensory nerve fibres and non-myelin Schwann cells are lacking, exhibit a disorganised vascular branching pattern with reduced maturation of the cutaneous vascular network [17]. In addition, the importance of non-myelin Schwann-cell-derived signals in the formation of vascular branching patterns has been demonstrated by its absence and the lack of concomitant peripheral nerve fascicles and blood vessels in the foetal skin of ErbB3 knockout mice. In contrast, it has been reported that in the foetal skin of Sema3A-deficient mice, where the cutaneous sensory nerve network is constructed in a disorganised manner, arterial vessels run parallel to the disorganised branching pattern of sensory nerve fibres [18]. In other words, the branching pattern of sensory nerve fascicles serves as a template for the branching pattern of blood vessels (Figure 2). The expression of chemokine CXCL12 from sensory nerve fascicles regulates arterial branching patterns in sensory nerve fibres. The action of secreted CXCL12 on CXCR4 receptors specifically expressed on vascular endothelial cells induces the migration of vascular endothelial cells of the adjacent atrial vascular plexus towards the sensory nerve fascicle. As indicated by the disruption of the parallel structure of peripheral nerve fascicles and blood vessels in the foetal skin of Cxcl12 and Cxcr4 knockout mice, CXCL12–CXCR4 signalling is involved in the regulation of vascular branching patterns by sensory nerve fascicles [19].

### 3.2. Regulation of Arterial Differentiation

It is believed that arteries and veins differentiate in response to physical stimuli produced by heartbeat and blood flow. However, the membrane-bound ligand ephrin B2 and its tyrosine kinase-type receptor EphB4, which are molecular markers in arteries and veins, are specifically expressed during the formation of the primary vascular plexus. Hence, it is possible that the fate of arteriovenous vessels is decided earlier than blood flow, although it has been demonstrated that the various signals received later are just as vital.

Activation of notch signalling by VEGFA is essential for arterial vascular endothelial cell differentiation [20]. The phospholipase C-γ (PLC-γ)-mitogen-activated protein kinase (MAPK) pathway, which is activated downstream of VEGFR2, is responsible for arterial differentiation [21]. VEGFA signalling affects arterial development by upregulating notch ligand Dll4 expression [22,23]. Upregulation of the Fox family transcription factors Foxc1 and Foxc2 is involved in artery formation by enhancing the expression of Dll4, Notch1, Notch4, and ephrin B2 [24]. Studies with knockout mice have demonstrated that Dll4-Notch1/4 signalling regulates Hey1/2 expression via RBP-J to influence arterial differentiation [25]. Furthermore, notch signalling has been shown to affect arteriogenesis by regulating CD36 expression [26].

During the development of arterioles in the skin, sensory-nerve-fascicle-derived “VEGF-A” promotes arterialisation of capillary endothelial cells by activating VEGFR2–neuregulin 1 receptor signalling (Figure 2). In sensory-nerve-fascicle-specific Vegfa knockout mice and vascular endothelium-specific neuropilin 1 knockout mice, arterial differentiation in the proximity of nerve fascicles is inhibited. It has been hypothesised that peripheral-nerve-derived VEGFA promotes arterial formation by increasing the sensitivity of vascular endothelial cells expressing VEGFR2 and NRP1 [19]. During the development of foetal mouse arteries, Cxcr4-expressing vascular endothelial cells are initially attracted by Cxcl12 produced by non-myelin Schwann cells in the sensory nerve fascicles. Subsequently, Vegfa surrounding the nerve fascicle induces arterialisation of attracted vascular endothelial cells. Thus, the coordinated action of Vegfa and Cxcl12 generates a branching pattern of arterial vessels that parallels peripheral sensory nerve fascicles.

### 3.3. Regulation of Venous Differentiation and Patterning

Although the mechanism of arterial endothelial cell differentiation has been elucidated, the specifics of venous endothelial cell differentiation remain unclear. The nuclear receptor COUPTF2 regulates venous endothelial cell differentiation by suppressing Notch signalling through NRP1 repression [27,28]. Consequently, venous determinants may acquire venous specificity by inhibiting arterial determinants. Furthermore, bone morphogenetic protein (BMP) signalling acts on venous differentiation by inducing EPHB4 expression through ALK3/BMPR1A receptors and SMAD1/SMAD5 signalling [29]. Arteriovenous differentiation is predicted to be regulated by specific factors and their identification is anticipated.

The branching pattern of veins that runs parallel to the arterial pattern develops after sensory nerve fascicles and arteries are formed. The arteriole–vein juxtaposition structure is formed by the production of the bioactive peptide apelin from the artery and its action on the apelin receptor (APJ) expressed in the vein (Figure 2). Activation of APJ increases the motility of vascular endothelial cells and promotes the secretion of Wnt-related protein SFRP1. This causes myeloid cells to produce matrix metalloproteinases, hence, degradation of collagen that anchors the veins causing it to migrate towards the arterial side [30]. Furthermore, DROSHA, a microRNA biosynthetic enzyme that regulates the TGF-β and BMP pathways via miRNAs, has been shown to be involved in the formation of venous branching patterns as its knockout mice demonstrate disrupted arterial and venous juxtaposition structures [31]. The counter-current heat exchange between adjacent arteries and veins enables thermostatic animals to adapt to environmental temperatures [32]. Owing to the interdependence of nerves and blood vessels, nerves define the vascular network, whereas blood vessels define the neural network. In either case, the wiring between the nerves and blood vessels is a result of their close relationship. Analysis of single-cell RNA sequencing data has recently offered molecular insights into arterial, capillary, and venous differentiation, and it is anticipated that this information can be used to advance our understanding of the relationship between nerves and arteriovenous development [33].

## 4. Neurovascular Interactions in Tumour Tissue

In addition to cancer cells, tumour tissues consist of blood vessels, nerves, immune cells, and extracellular matrix. Their characteristics are determined by the interaction between cancer cells and the tumour microenvironment (TME) [34]. Developmental organogenesis and vascularisation are dependent on the nerves [35]. Therefore, it is possible that a similar mechanism occurs during tumour progression, in which nerves stimulate angiogenesis and promote tumour growth through the secretion of angiocrine factors by endothelial cells.

**Figure 3:**
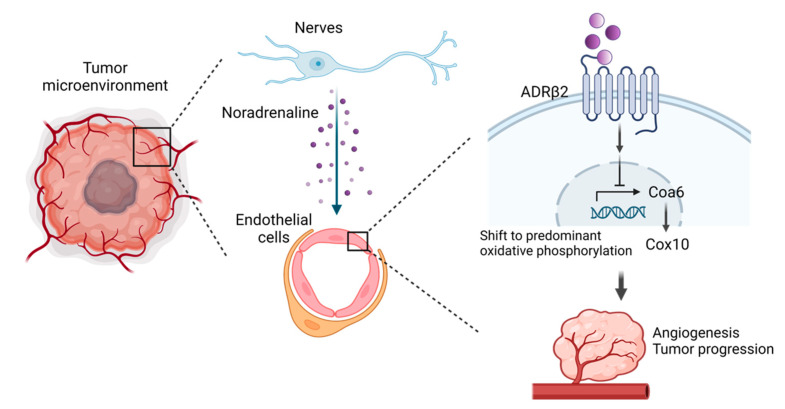
Interactions of neuro-vascular system in the tumour microenvironment (TME). In the TME, neuronal factors promote angiogenesis and tumour growth by acting on vascular endothelial cells. In prostate cancer, noradrenaline secreted by the autonomic nervous system regulates metabolic activity by acting on β2-adrenergic receptors (Adrb2) on vascular endothelial cells. The noradrenergic signalling regulates Coa6 transcription; increased Coa6 expression results in a Cox10-dependent metabolic bias towards oxidative phosphorylation in endothelial cells. Adr2 was highly expressed on vascular endothelial cells in highly malignant tumours. Created with BioRender.com.

Indeed, numerous solid tumours have enlarged nerves, and increased nerve density is related to tumour growth and prognosis [36,37]. Autonomic innervation regulates tumour development and dissemination [38]. In the central nervous system, neural progenitor cells that express doublecortin (DCX) infiltrate prostate cancer and metastases and initiate neurogenesis [39]. This is achieved through the oscillation of DCX+ neural progenitor cells in the subventricular zone, which results in the disruption of the blood–brain barrier and efflux of DCX+ cells into the bloodstream, where they infiltrate and colonise the tumour and generate nascent neurons. Recent research has also revealed that peripheral nerves interact with tumour and stromal cells to promote the development and progression of various solid tumours [40]. With respect to the direct interaction between nerves and blood vessels, autonomic nerves have been shown to induce pathological vascular networks and promote prostate cancer [41]. In a mouse model, β2 adrenergic receptor (Adrb2) and Adrb3 knockout stromal cells in the TME significantly inhibited the proliferation of prostate cancer. The adrenergic nerves of the exchange nervous system release noradrenaline and transmit signals via α-adrenergic and β-adrenergic receptors on the target cells [42]. Noradrenaline secreted by neurons acts on endothelial cells expressing Adrb2 to regulate their metabolism (Figure 3). Tumour regression coincides with a decrease in the number of blood vessels, and Adrb2 is highly expressed in endothelial cells, particularly in high-grade tumours. 

Contrary to expectations, clinical trials of angiogenesis inhibitors in cancer therapy have not proven survival extension [43]. Several resistance mechanisms to angiogenesis inhibitors have been reported, some of which have been related to neurology [44]. Among the proposed resistance mechanisms there is a process known as vascular co-option, in which tumour cells lacking vascular supply incorporate normal blood vessels present in nearby normal tissues [45]. Co-option allows diffusely infiltrating tumour cells to acquire blood flow in astrocytomas without angiogenesis [46]. In other words, angiogenesis inhibitors have no antitumor effects and fail to suppress co-option. It is most prevalent in cancers of the brain, lungs, and liver, and is abundant in blood vessels [47]. 

Interactions between neurons and blood vessels have also been suggested to be involved in tumour metastasis. L1 cell adhesion molecule (L1CAM), which is essential for neurogenesis processes, such as neuronal migration and differentiation, functions as an adhesion factor for metastatic cells as they adhere to and migrate along blood vessels [48]. As a result of cancer cells leaving blood vessels, astrocytes produce tissue plasminogen activator, which generates plasmin from neuron-derived plasminogen and mobilises FasL from astrocytes, thereby killing cancer cells. Plasmin cleaves and inactivates L1CAM, a protein that facilitates adhesion of cancer cells to blood vessels. Conversely, metastasis expands when tumour cells express Plasminogen Activator Inhibitor-1, which inhibits tumour cell survival and vascular adhesion [49]. Thus, it has been hypothesised that nerves and blood vessels in tumour tissues interact to determine the characteristics of cancer, and elucidating the crosstalk between nerves and blood vessels around tumour cells could provide clues for the development of new cancer treatments.

## 5. Neurovascular Interactions in Tissue Repair

Tissue repair systems are triggered automatically when a tissue is damaged. Blood vessels play an essential role in the wound healing process and new blood vessels are formed at the site of injury. For example, in bedsores, capillaries on the body surface are continuously compressed, resulting in poor blood flow to the skin and ischemic necrosis. Therefore, the use of a therapeutic agent that promotes angiogenesis as a treatment for bedsores promotes granulation tissue formation and repair [50]. Thus, there is no doubt that angiogenesis is critical for the tissue repair of bedsores. It is becoming clear that angiogenesis is critical not only for wound healing of normal tissues such as skin, but also for functional recovery of the central nervous system, a difficult organ to repair. The vascular system has been reported to be closely related to the nervous system [51]. Although the central nervous system is isolated from the external environment by the blood–brain barrier and blood–cerebrospinal fluid barrier, in central nervous system diseases, inflammation is triggered at the lesion site, the vascular barrier is disrupted, and a pathological condition is formed. The role of vasculature in the repair of neural circuits is unclear, but recent studies have provided insights into the mechanisms involved. Molecules such as VEGF [52,53], which stimulates angiogenesis during cerebral ischemia, and prostacyclin [54] and apelin [55], which are secreted by vascular endothelial cells, have also been discussed as contributing to neural repair. Several molecules have also been discussed.

Therapy for cerebral infarction and other cerebrovascular diseases is vital to establish appropriate measures in order to recover the function of the sequelae after disease onset. The current treatment strategy to restore neurological function is to promote angiogenesis, as in normal tissues, such as the skin [56]. In a rat model of transient focal cerebral ischemia, systemic administration of VEGF at 48 h after onset promoted angiogenesis in the ischemic area and significantly restored neural function. In stark contrast, systemic administration of VEGF 1 h after ischaemia exacerbated BBB leakage and haemorrhagic changes due to increased vascular permeability [57]. The role of VEGF in tissue repair during cerebral ischemia is dependent on the period of stimulation or inhibition, indicating that angiogenesis is significantly involved in the exacerbation of neural repair. Angiogenesis has also been shown to be involved in the repair of injured nerve function, not only in cerebral infarction but also in spinal cord injury models that cause secondary acute focal ischemia. It has also been shown that the administration of VEGF to this pathological model promotes angiogenesis in the ischaemic area, decreases neuronal apoptosis, and helps maintain function.

**Figure 4:**
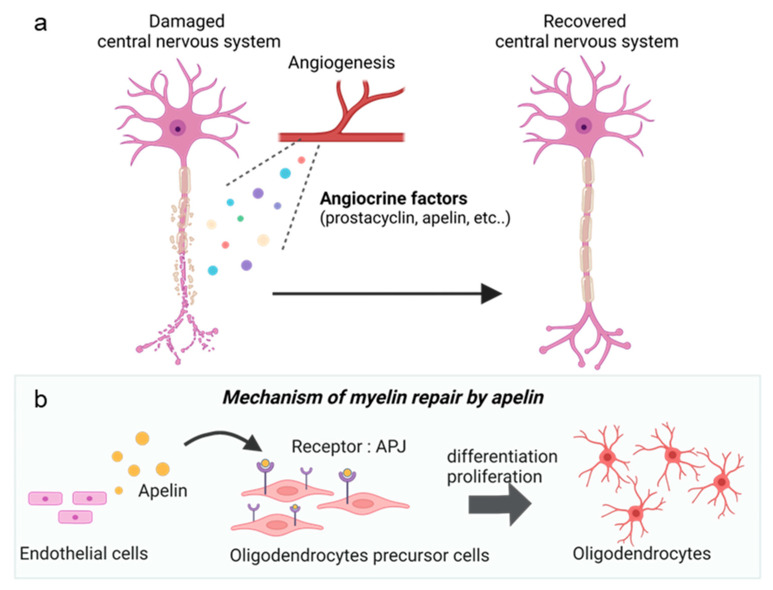
Nerve repair by angiocrine factors. (**a**) Damaged nerves are accompanied by angiogenesis during repair. Lipids and peptides such as prostacyclin and apelin are secreted by vascular endothelial cells from the formed blood vessels to promote nerve repair. (**b**) Apelin is secreted from blood vessels formed in the vicinity of the damaged nerve. Secreted apelin binds to and activates APJ receptors expressed on oligodendrocyte precursor cells, thereby promoting differentiation into oligodendrocytes. Differentiation into oligodendrocytes and myelination are promoted. Created with BioRender.com.

Recent studies have provided detailed mechanisms for the interaction between the blood vessels and nerves during tissue repair. Angiocrine factors such as prostacyclin and thromboxane [58] secreted from vascular endothelial cells have been shown to function during nerve repair (Figure 4). Prostacyclin is a member of the prostaglandin family that inhibits thrombus formation by suppressing platelet activation and vasodilatory effects. In an experimental autoimmune encephalomyelitis model of multiple sclerosis, prostacyclin has been shown to initiate angiogenesis in the neighbourhood of the lesion; it is secreted by the endothelial cells of the lesion to promote neurite outgrowth. Prostacyclin acts on type I prostaglandin receptors (IP receptors) in neurons and increases the production of cAMP, which promotes neurite extension and contributes to the repair of neuronal function. In addition to angiocrine factors, there have been studies on blood components derived from blood vessels that contain molecules that promote repair [59]. The hormone-like substance FGF21, a member of the fibroblast growth factor family, is essential for angiogenesis, wound healing, and embryonic development. FGF21 is secreted from the pancreas and contributes to repair during myelin sheath injury by promoting the proliferation of oligodendrocyte progenitor cells in lesions at sites of vascular barrier disruption and influx. In addition, injection of Lysophospatidylcholin into FGF21-deficient mice to induce demyelination resulted in the suppression of myelin sheath repair and absence of improvement in motor function associated with myelin sheath repair compared to control mice [60]. Hence, it has been suggested that the blood-mediated linkage between the central nervous system and end organs contributes to the repair of neural circuits.

It has also been reported that a decrease in angiocrine factor secretion, associated with aging, affects neural repair. To identify factors that reduce the differentiation ability of oligodendrocytes with aging, molecular expression patterns among immature oligodendrocytes, progenitors of cultured oligodendrocytes, and differentiated oligodendrocytes in young and old mice were explored, where the expression of APJ was found to be higher in oligodendrocytes with high differentiation ability compared to other oligodendrocytes. Furthermore, to elucidate the effect of the APJ on myelination in vivo, mice lacking the APJ specific for oligodendrocytes were analysed. As a result, myelination defects were observed in the corpus callosum, and motor function deteriorated compared to controls. Since the ligand for APJ, apelin, has also been shown to decrease with aging, it has been suggested that activation of the apelin-APJ promotes oligodendrocyte differentiation and contributes to the repair of the myelin sheath (Figure 4). Hence in a study, demyelination was induced in aged mice, and administration of a compound active on the APJ resulted in significant recovery of the myelin sheath as well as recovery of locomotor function.

In the experimental autoimmune encephalomyelitis and aging models, administration of a compound acting on the APJ was found to improve the pathophysiology of the disease. These results may lead to improved brain function in the elderly and the development of treatments for demyelinating diseases such as multiple sclerosis.

Neuronal networks (neural circuits) maintain brain function. Hence, damage to the brain due to various causes can injure neural circuits, resulting in loss of function and symptoms of cranial nerve diseases. It is therefore imperative to repair the damaged neuronal circuits to preserve cerebral capacity. Recent studies have revealed that the repair of neuronal circuits is regulated not only by cells of the nervous system but also by cells of the immune, endocrine, and vascular systems. The significance of angiocrine factors during neural repair is described here as well as in other studies, paving the way to identify angiocrine factors that accelerate aging, that is, those that lead to pathogenesis, for future drug discovery. Hence, we can look forward to the development of therapeutic agents targeting angiocrine factors for cranial nerve diseases.

## 6. Perspective

Almost all tissues develop blood vessels, which are fundamental units of physiological activity. Although blood vessels appear to be simple tissues composed of vascular endothelial cells and pericytes, they develop into those that are tailored to each of the various organs through dynamic changes brought about by interactions with the surrounding cells. In particular, there is close communication between the peripheral nerves and blood vessels, forming a tissue-specific and functional network of nerves and blood vessels. By utilising new analytical techniques, such as in vivo imaging and single-cell omics, a comprehensive view of ‘neurovascular interactions’, which participate in various aspects of biological processes such as ontogeny, homeostasis, disease, and tissue regeneration, is expected.

## Data Availability

Not applicable.
